# Supplementary figures and images for: Functional prediction of proteins from the human gut archaeome
Source: ISME Commun. 2024 Jan 10;4(1):ycad014. doi: 10.1093/ismeco/ycad014 (PMC10939349; doi:10.1093/ismeco/ycad014)

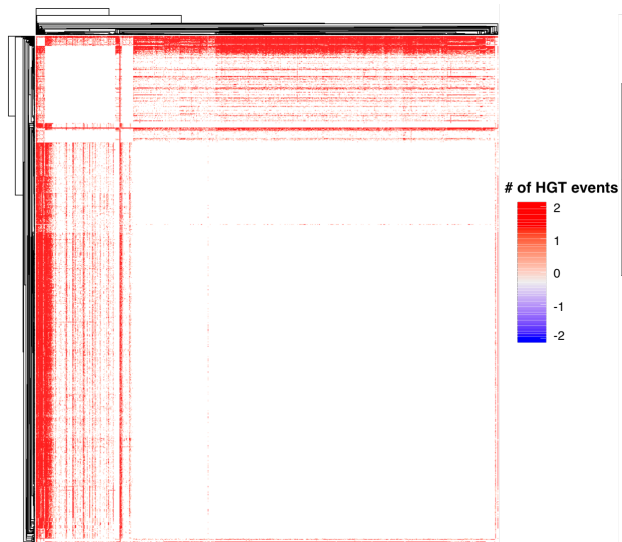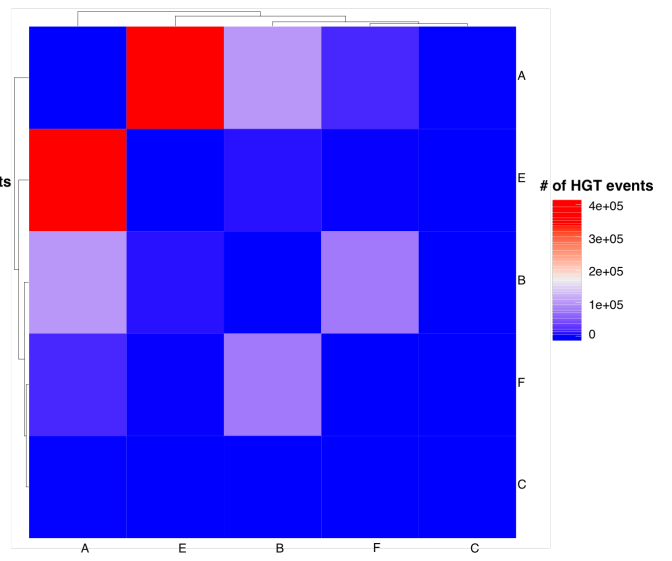

Supplement: supp_fig_1_ycad014 [file supp_fig_1_ycad014.pdf]

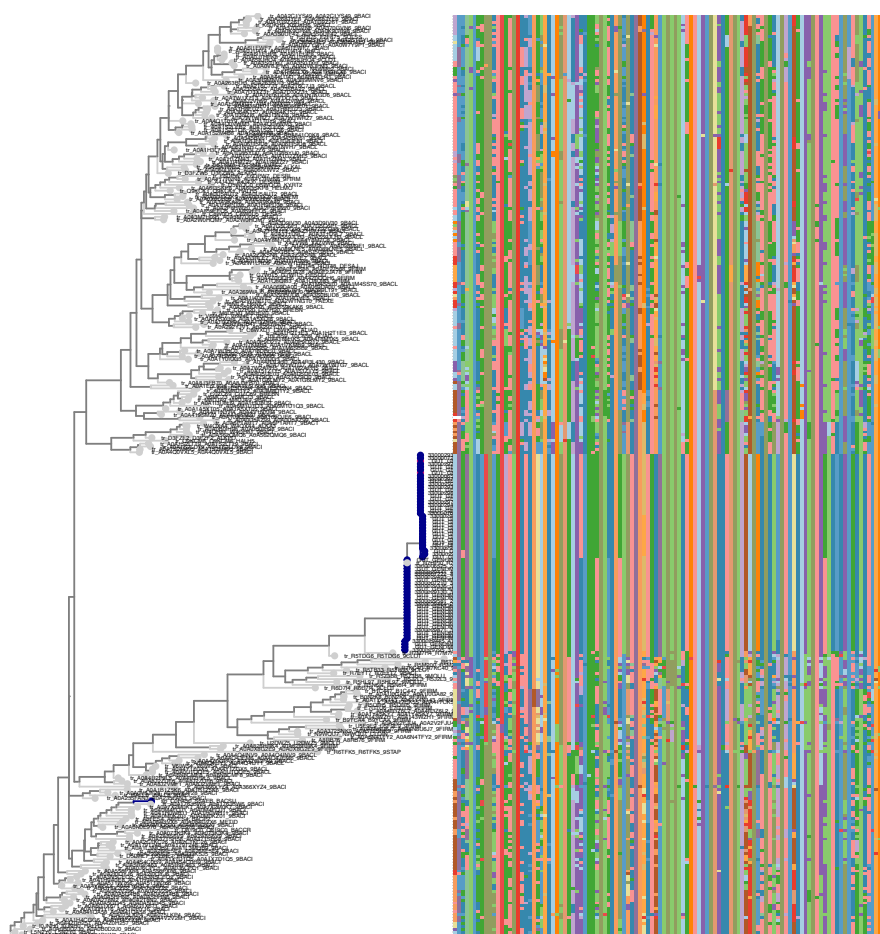

seq

- m  
a n  
c p  
d q  
e r  
f s  
g t  
h v  
i w  
k y  
l

taxa

• GUT\_archaea  
• GUT\_bacteria  
• uniprot

Supplement: supp_fig_2_ycad014 [file supp_fig_2_ycad014.pdf]

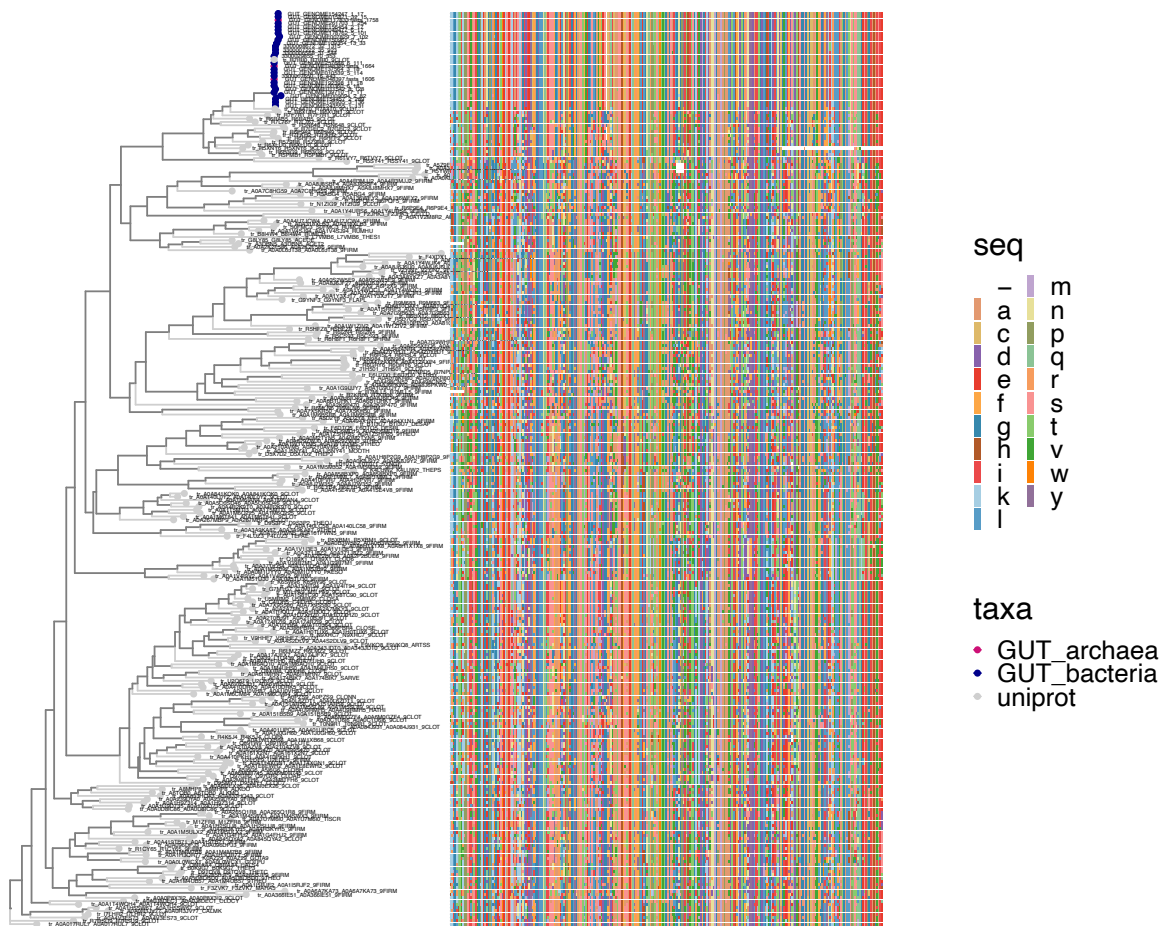

Supplement: supp_fig_3_ycad014 [file supp_fig_3_ycad014.pdf]

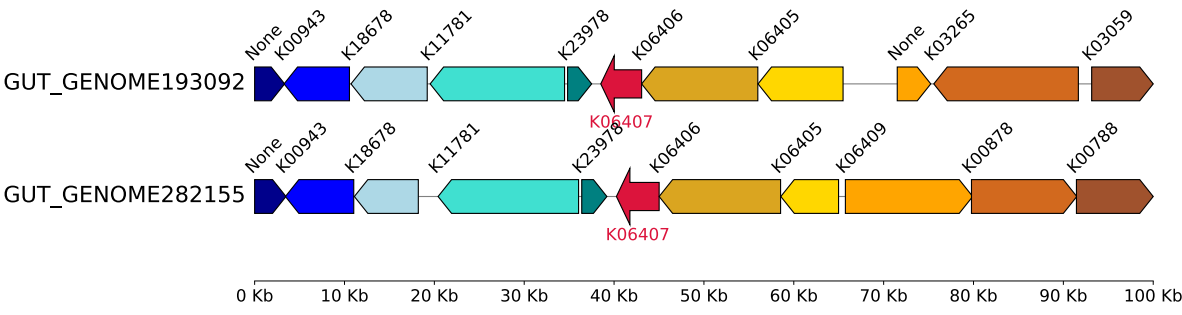

Supplement: supp_fig_4_ycad014 [file supp_fig_4_ycad014.pdf]

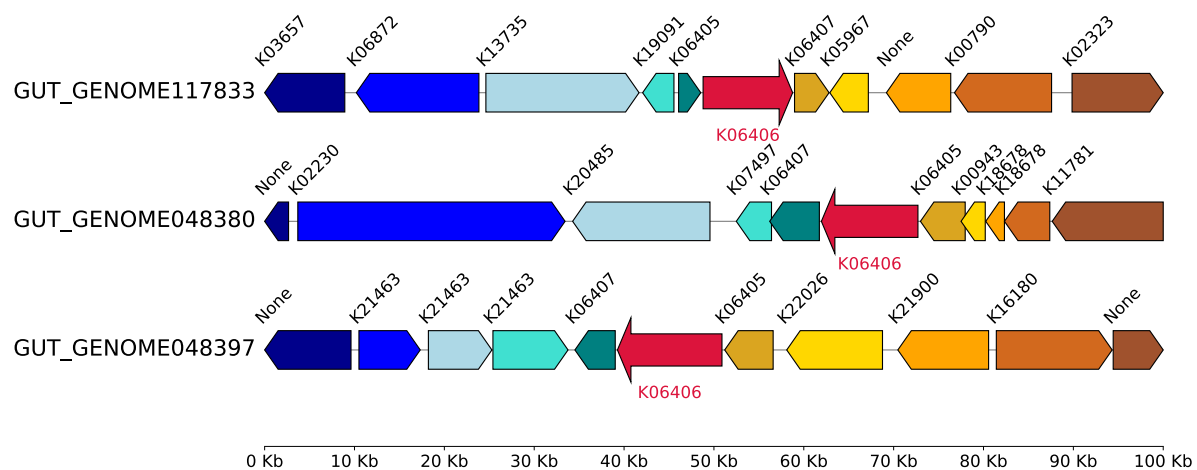

Supplement: supp_fig_5_ycad014 [file supp_fig_5_ycad014.pdf]
