# Supplementary material for: Functional prediction of proteins from the human gut archaeome
Source: ISME Commun. 2024 Jan 10;4(1):ycad014. doi: 10.1093/ismeco/ycad014 (PMC10939349; doi:10.1093/ismeco/ycad014)
Supplement: Supp_tab_and_fig_legends_ycad014 [file supp_tab_and_fig_legends_ycad014.docx]

***Table 1.*** *The summary of annotations provided by trRosetta, AlphaFold2.0-based ProFunc report, and DeepFRI for unique archaeal proteins*

***Table 2.*** *The summary of annotations provided by trRosetta, AlphaFold2.0-based ProFunc report, and DeepFRI for homologous archaeal proteins*

***Table 3.*** *Proportion of proteins modelled with AlphaFold2 and trRosetta with respective confidence scores.*

***Table 4.*** *Sequence- and structure-based annotations of unique archaeal gut-specific proteins.*

***Table 5.*** *Sequence- and structure-based annotations of homologous archaeal gut-specific proteins.*

***Table 6.*** *Sequence similarity between unique archaeal gut-specific proteins and UniProt sequences corresponsing to sequence-based functional annotations.*

***Table 7.*** *Sequence similarity between homologous archaeal gut-specific proteins and UniProt sequences corresponsing to sequence-based functional annotations.*

***Table 8.*** *Accuracy estimation for structure-based functional assignment over sequence-based methods for unique and homologous protein clusters.*

***Figure 1****. Heatmaps demonstrating the intensity of HGT events between M. smithii genomes. A, HGT between taxonomic groups named as follows: A - Methanobrevibacter_A smithii, B - Methanobrevibacter_A smithii_A (Ca. Methanobreviabcter intestini), C - Methanobrevibacter_A oralis, E - GCF_000016525.1 (M. smithii), F - GCF_002252585.1 (Ca. Methanobreviabcter intestini); B, HGT events between individual genomes of same groups. The legend depicts the frequency of HGT events among the genomes of A, taxonomic groups and B, individual genomes.*

***Figure 2****. Phylogenetic tree of stage V sporulation proteins AE from identified SC h9 and Uniprot. Bacterial and archaeal proteins from cluster h9 are depicted as GUT_bacteria and GUT_archaea in dark blue and pink, respectively.*

***Figure 3.*** *Phylogenetic tree of stage V sporulation proteins AD from identified SC h20 and Uniprot. Bacterial and archaeal proteins from cluster h9 are depicted as GUT_bacteria and GUT_archaea in dark blue and pink, respectively.*

***Figure 4.*** *Gene synteny of homologous bacterial sequences obtained from the human gut dataset that share similarities with the archaeal sequences from cluster h9 encoding stage V sporulation protein AE (spoVAE).*

***Figure 5.*** *Gene synteny of homologous bacterial sequences obtained from the human gut dataset that share similarities with the archaeal sequences from cluster h20 encoding stage V sporulation protein AD (spoVAD).*
